# Lower Urinary Tract Symptoms and Female Sexual Function During Pregnancy: A Correlational and Comparative Analysis

**DOI:** 10.3390/healthcare14193267

**Published:** 2026-10-02

**Authors:** Ahmet Şanlı, Nuran Nur Aypar Akbağ, Yasemin Şanlı

**Affiliations:** 1 Department of Urology, Faculty of Medicine, Karamanoğlu Mehmetbey University, 70200 Karaman, Türkiye; drasanli07@gmail.com; 2 Department of Midwifery, Faculty of Health Sciences, Sinop University, 57010 Sinop, Türkiye; nuraypar@gmail.com; 3 Department of Midwifery, Faculty of Health Sciences, Karamanoğlu Mehmetbey University, 70200 Karaman, Türkiye

**Keywords:** pregnancy, lower urinary tract symptoms, female sexual function, sexual dysfunction

## Abstract

**Aim:** This study aimed to examine the relationship between lower urinary tract symptoms and sexual function among pregnant women and to evaluate variations in these outcomes according to demographic, obstetric, and sexual characteristics. **Methods:** This descriptive, cross-sectional, and correlational study was conducted with 278 pregnant women. Data were collected through face-to-face interviews between June and July 2026. The data collection instruments included the Descriptive Information Form, the Bristol Female Lower Urinary Tract Symptoms Questionnaire (BFLUTS), and the Female Sexual Function Index (FSFI). **Results:** The mean age of the pregnant women was 28.88 ± 4.13 years. The mean total FSFI score was 19.06 ± 5.72, with 95.7% of participants scoring below the conventional cutoff of 26.55. Women scoring below the conventional FSFI cutoff had significantly higher BFLUTS filling and urinary incontinence scores. In the adjusted regression model, greater urinary filling symptom severity (B = −0.346, SE = 0.162, β = −0.145, t = −2.129, *p* = 0.034) and a history of curettage (B = 2.436, SE = 1.088, β = 0.160, t = 2.238, *p* = 0.026) were associated with lower total FSFI scores. Obstetric characteristics, self-reported sexual knowledge, and sexual practices were also associated with variations in specific urinary symptom and sexual function domains. **Conclusions:** Urinary filling symptoms and obstetric history were associated with sexual function during pregnancy, even after adjustment for other characteristics. These findings support the consideration of urinary symptoms and sexual health in comprehensive antenatal assessment and highlight the potential value of individualized counseling.

## 1. Introduction

Pregnancy is accompanied by substantial hormonal, anatomical, and biomechanical changes that may affect lower urinary tract and pelvic floor function. Increased uterine volume, mechanical loading of the pelvic floor, hormonal influences on smooth muscle and connective tissue, and alterations in pelvic floor support may contribute to the development of lower urinary tract symptoms (LUTS), including storage, voiding, post-micturition, and urinary incontinence symptoms [1,2]. LUTS are highly prevalent during pregnancy and may become more pronounced as gestation progresses [3,4,5]. Recent evidence from a large sample of pregnant women showed that 99.2% experienced at least one LUTS, with storage symptoms being particularly common [5]. These findings indicate that LUTS constitute an important component of maternal health that warrants attention during antenatal care.

Pregnancy may also substantially affect female sexual function. Physiological and hormonal changes, altered body image, physical discomfort, pregnancy-related concerns, and psychosocial factors may influence sexual desire, arousal, lubrication, orgasm, satisfaction, and pain [6,7,8,9]. Previous studies have demonstrated changes in sexual activity and sexual function across pregnancy, with poorer sexual function particularly evident in later gestation in some populations [8,9]. A recent systematic review further showed that sexual desire fluctuates throughout pregnancy and emphasized the contribution of both physiological and psychological factors to pregnancy-related changes in sexuality [10].

Although both LUTS and alterations in sexual function are common during pregnancy, their interrelationship has received comparatively less attention. Existing pregnancy-specific research has largely examined LUTS in terms of prevalence, severity, associated factors, or quality-of-life consequences [3,4,5], while studies of sexual function have predominantly focused on gestational changes and demographic, obstetric, psychological, and relationship-related factors [6,7,8,9,10]. Moreover, previous prospective research examining sexual function alongside pelvic floor symptoms primarily focused on changes across pregnancy and the postpartum period rather than specifically characterizing the relationships between multidimensional LUTS and different domains of sexual function during pregnancy [11].

Understanding the relationship between LUTS and sexual function during pregnancy is important for a comprehensive understanding of maternal urinary and sexual health. Although previous studies have examined urinary symptoms and sexual function during pregnancy, evidence regarding the associations between specific LUTS domains and different aspects of sexual function remains limited, particularly when demographic, obstetric, and pregnancy-related sexual characteristics are considered simultaneously. Therefore, this study aimed to investigate the relationships between specific LUTS domains (filling, voiding, and urinary incontinence) and female sexual function among pregnant women, to examine variations in these outcomes according to demographic, obstetric, and pregnancy-related sexual characteristics, and to determine whether LUTS are independently associated with sexual function after accounting for relevant covariates. By simultaneously evaluating multidimensional LUTS and domain-specific sexual function using validated instruments, this study seeks to provide a more integrated understanding of urinary and sexual health during pregnancy and to contribute evidence that may support comprehensive assessment and counseling in antenatal care.

## 2. Materials and Methods

### 2.1. Study Design, Setting and Participant

This study was designed as a descriptive, cross-sectional, and correlational study. It was conducted between June and July 2026 with pregnant women who attended the obstetrics outpatient clinics of Karaman Training and Research Hospital, located in the Central Anatolia Region of Türkiye, and agreed to participate in the study. The study was reported in accordance with the Strengthening the Reporting of Observational Studies in Epidemiology (STROBE) guidelines.

The minimum required sample size was calculated using the single-population proportion formula for an unknown population size. Based on the prevalence of lower urinary tract symptoms among pregnant women reported by Balik et al. (81.6%) [12], with a 95% confidence level (Z = 1.96) and a 5% margin of error (d = 0.05), the minimum required sample size was calculated as 231 pregnant women [*n* = Z^2^*p*(1 − *p*)/d^2^]. A convenience sampling method was used, and eligible pregnant women who attended the obstetrics outpatient clinics during the data collection period and agreed to participate were recruited. The study was completed with 278 pregnant women.

The inclusion criteria were being able to read and write, being in the second or third trimester of pregnancy (pregnant women in the first trimester were excluded because they may experience sexual dysfunction [9,12,13]), being in a regular relationship with a partner, not having a high-risk pregnancy requiring restriction of sexual activity as advised by a physician, and providing voluntary informed consent to participate. Women who self-reported a diagnosed psychiatric disorder, cognitive impairment, communication difficulties, or anatomical or functional disorders of the lower urinary tract were excluded from the study. In addition, participants who did not complete the data collection forms were excluded from the final analysis. These exclusion criteria were assessed based on participants’ self-reports.

### 2.2. Instruments

Data were collected using the Descriptive Information Form, the Bristol Female Lower Urinary Tract Symptoms Questionnaire (BFLUTS), and the Female Sexual Function Index (FSFI).

Descriptive Information Form: The form was developed by the researchers based on the relevant literature [3,4,6]. It included questions on participants’ sociodemographic and obstetric characteristics, such as age, educational level, employment status, family type, income level, pregnancy intention, and pregnancy-related health problems.

The Bristol Female Lower Urinary Tract Symptoms Questionnaire (BFLUTS): The BFLUTS was developed by Jackson et al. in 1996 [14], and its Turkish validity and reliability were established by Gökkaya et al. in 2012 [15]. The questionnaire was designed to assess urinary incontinence, other lower urinary tract symptoms, sexual health, and health-related quality of life. It consists of 19 items grouped into five subscales: filling (Items 1–4), voiding (Items 5–7), urinary incontinence (Items 8–12), sexual function (Items 13–14), and quality of life (Items 15–19). Items 4, 13, 14, 17, and 19 are scored on a 4-point Likert scale (0–3), whereas the remaining items are scored on a 5-point Likert scale (0–4). Total scores range from 0 to 71, with higher scores indicating greater severity of LUTS and a greater negative impact on sexual life and quality of life [15]. Only the filling, voiding, and urinary incontinence subscales of the BFLUTS were included in the regression analyses. The sexual function and quality-of-life subscales were excluded to maintain the focus on urinary symptoms and to minimize potential conceptual overlap with the FSFI, which was used as the primary outcome measure. In the present study, the Cronbach’s alpha coefficient for the total BFLUTS was 0.81.

Female Sexual Function Index (FSFI): The FSFI was developed by Rosen et al. to assess female sexual function [16]. It evaluates sexual function and sexual problems experienced during the previous four weeks. The FSFI is a multidimensional instrument consisting of 19 items and six subscales: desire, arousal, lubrication, orgasm, satisfaction, and pain. The Turkish version was validated and found to be reliable by Aygin and Aslan [17]. Subscale scores are multiplied by their respective factor weights, resulting in a total score ranging from 2 to 36, with higher scores indicating better sexual function. A total score of 26.55 is accepted as the cutoff value for female sexual dysfunction [17]. Although this cutoff was originally developed for the general female population, it has been widely applied in studies evaluating pregnant populations to classify sexual dysfunction and estimate its prevalence [18,19]. In the Turkish validation study, the Cronbach’s alpha coefficient was reported as 0.98. In the present study, the Cronbach’s alpha coefficient for the total FSFI was 0.90.

### 2.3. Data Collection

Before data collection, participants were informed about the purpose and scope of the study. They were also informed that participation was entirely voluntary, and written informed consent was obtained from all participants. Data were collected through face-to-face interviews. The entire data collection process took approximately 20–25 min for each participant.

### 2.4. Statistical Analysis

The data were analyzed using IBM SPSS Statistics version 25.0 (IBM Corp., Armonk, NY, USA). Descriptive statistics were presented as frequencies and percentages for categorical variables and as means, standard deviations, and minimum–maximum values for continuous variables. Skewness and kurtosis coefficients (ranging from −2 to +2) were analyzed to assess normality, and the results indicated a normal distribution [20]. Since all variables were found to be normally distributed, parametric tests were applied.

Pearson’s correlation analysis was conducted to examine the relationships between specific lower urinary tract symptom dimensions (filling, voiding, and urinary incontinence) and female sexual function subscales. The 21 correlations were considered exploratory analyses. To account for multiple testing within this family of correlations, *p* values were adjusted using the Benjamini–Hochberg false discovery rate (FDR) procedure. Both unadjusted *p* values and FDR-adjusted *p* values (q values) are reported, with q < 0.05 considered statistically significant after correction. A hierarchical multiple linear regression analysis was performed to evaluate the multivariate associations between lower urinary tract symptoms and female sexual function after controlling for demographic, obstetric, and pregnancy-related sexual characteristics. In the hierarchical multiple linear regression analysis, unstandardized coefficients (B), standard errors (SE), standardized regression coefficients (β), 95% confidence intervals (95% CI), R^2^, adjusted R^2^, changes in R^2^ (ΔR^2^), F-change statistics, and their corresponding *p* values were reported. Regression assumptions were assessed before interpreting the models. Multicollinearity was evaluated using variance inflation factor (VIF) and tolerance values, with VIF < 5 and tolerance > 0.20 considered acceptable [21]. Variables were selected for inclusion in the hierarchical regression models based on their statistically significant correlations with total FSFI scores and their established clinical and theoretical relevance to sexual health. Marriage duration, trimester, parity, and knowledge of sexuality during pregnancy were also included despite their nonsignificant correlations with total FSFI scores [6,8,22]. Variables were entered in three sequential blocks: BFLUTS subscale scores (Filling, Voiding, and Urinary Incontinence) in Model 1; sociodemographic and obstetric characteristics in Model 2; and pregnancy-related sexual characteristics in Model 3. This sequence was used to examine the associations between lower urinary tract symptoms and sexual function and to assess the incremental explanatory contribution of the subsequent variable blocks. Independent-samples t-tests and one-way analysis of variance (ANOVA) were used to compare symptom severity and sexual function scores according to demographic, obstetric, and pregnancy-related sexual characteristics. Cohen’s d and eta squared (η^2^) were calculated as effect sizes for two-group comparisons and ANOVA, respectively. To control for the increased risk of Type I error due to multiple testing across the 10 BFLUTS and FSFI outcomes, a Bonferroni-adjusted significance threshold of *p* < 0.005 (0.05/10) was applied to these comparisons [21]. For ANOVA results meeting this adjusted significance criterion, pairwise comparisons were performed using the Bonferroni post hoc procedure, and Bonferroni-adjusted *p* values of <0.05 were considered statistically significant.

### 2.5. Ethical Approval

Ethical approval for the study was obtained from the Karamanoğlu Mehmetbey University Health Sciences Scientific Research and Publication Ethics Committee (Date: 18 May 2026; Approval No. 03-2026/70). Institutional permission to conduct the study was obtained from Karaman Training and Research Hospital, where the study was conducted. The study was carried out in accordance with the ethical principles of the Declaration of Helsinki. Written informed consent was obtained from all participants prior to their enrollment in the study.

## 3. Results

The mean age of the pregnant women was 28.88 ± 4.13 years (range: 18–41 years). More than half of the participants (56.8%) had attained a university degree or higher. The mean duration of marriage was 5.22 ± 4.19 years. Most of the women were in the third trimester of pregnancy (74.5%), and 39.6% were primigravida. More than half of the participants were nulliparous (52.2%, *n* = 145). In addition, 84.2% reported having knowledge about sexuality during pregnancy, and 33.8% reported having sexual intercourse once every two weeks during the current pregnancy (Table 1).

The mean BFLUTS and FSFI scores of the pregnant women are presented in Table 2. The BFLUTS subscale means scores for filling, voiding, urinary incontinence, Sexual Function and Quality of Life were 6.60 ± 2.40, 1.49 ± 1.73, 2.83 ± 3.06, 0.44 ± 0.85, and 4.06 ± 3.07 respectively. The FSFI mean scores for desire, arousal, lubrication, orgasm, satisfaction, and pain were 3.95 ± 0.93, 3.22 ± 1.42, 3.17 ± 1.44, 3.08 ± 1.48, 2.07 ± 1.32, and 3.58 ± 1.86, respectively, with a total score of 19.06 ± 5.72. Additionally, 95.7% of the participants scored below the traditional threshold of 26.55 (Table 2).

Correlation analyses between the FSFI and BFLUTS scores were adjusted for multiple testing using the Benjamini–Hochberg false discovery rate procedure. After FDR correction, only the correlation between the FSFI Arousal subscale and the BFLUTS Voiding subscale remained statistically significant (r = 0.196, *p* = 0.001, q = 0.021), indicating a weak positive association. None of the other correlations remained statistically significant after FDR correction (q > 0.05) (Table 3).

As presented in Table 4, factors associated with female sexual function were examined using hierarchical multiple linear regression analysis. Model 1, which included the BFLUTS filling, voiding, and incontinence subscales, was not found to be statistically significant (R^2^ = 0.004; F(3.274) = 0.349; *p* = 0.790). In Model 2, where sociodemographic and obstetric variables were added, the explained variance rose to 10.7%, and a significant increase of 10.3% in explained variance was observed (ΔR^2^ = 0.103; ΔF(11,263) = 2.756; *p* = 0.002). In Model 2, a history of curettage was found to be significantly associated with the FSFI total score. In Model 3, which included variables related to sexual life during pregnancy, the total explained variance reached 13.7%, but the additional contribution of the final block was not statistically significant (ΔR^2^ = 0.030; ΔF(4.259) = 2.256; *p* = 0.064). The final model as a whole is significant (F(18,259) = 2.281; *p* = 0.003; adjusted R^2^ = 0.077). In this model, a history of curettage and the BFLUTS Filling subscale were found to be significantly associated with the FSFI total score (Table 4).

After Bonferroni correction, BFLUTS Filling scores were significantly higher in the third trimester than in the second trimester (t = −4.231, *p* < 0.001, d = 0.584). There was a significant difference between gravidity groups regarding urinary incontinence (t = −3.124, *p* = 0.002, d = 0.381) and FSFI Satisfaction scores (t = 2.990, *p* = 0.003, d = 0.367). Urinary incontinence scores also differed significantly by parity (F = 13,946, *p* < 0.001, η^2^ = 0.092). Bonferroni-adjusted post hoc comparisons showed that multiparous women had significantly higher urinary incontinence scores than nulliparous and primiparous women. Primiparous women also had higher scores than nulliparous women. Curettage experience was associated with differences in FSFI Lubrication, Orgasm, Satisfaction, Pain, and Total scores, but not in the BFLUTS subscale scores (Table 5).

Women who reported having knowledge about sexuality during pregnancy had higher BFLUTS Filling scores than those who did not (t = 3.818, *p* < 0.001, d = 0.628). Bonferroni post hoc comparisons showed that women reporting intercourse three or more times per week had significantly higher Filling scores than those reporting intercourse twice a week and once every two weeks. Intercourse frequency during pregnancy was associated with BFLUTS Urinary Incontinence (F = 8.773, *p* < 0.001, η^2^ = 0.088), FSFI Orgasm (F = 7.196, *p* < 0.001, η^2^ = 0.073), and FSFI Pain scores (F = 7.293, *p* < 0.001, η^2^ = 0.074). Bonferroni post hoc analysis showed higher urinary incontinence scores in women reporting intercourse three or more times per week than in all other groups. Women reporting intercourse twice a week had higher FSFI Orgasm scores than those reporting intercourse once every two weeks. Also, women reporting intercourse twice a week had higher FSFI Pain scores than women reporting intercourse once a week or once every two weeks. Finally, avoidance of sexual intercourse during pregnancy was associated with differences in FSFI Desire, Lubrication, Orgasm, and Pain scores (Table 5).

## 4. Discussion

This study aimed to examine LUTS and female sexual function among pregnant women, evaluate their variations according to demographic, obstetric, and sexual characteristics, and investigate the associations between LUTS dimensions and female sexual function after adjustment for relevant covariates.

The findings of this study indicate that lower urinary tract symptoms were common among pregnant women, with filling symptoms being particularly prominent. Sexual function was also affected across several domains, including satisfaction and orgasm, and a substantial proportion of participants scored below the conventional FSFI threshold. Similar findings have been reported in recent studies, indicating that impaired sexual function is common during pregnancy and may vary across gestation [10,19,22]. These changes may reflect the combined influence of physiological, psychological, and relationship-related factors accompanying pregnancy [10]. However, the conventional FSFI cutoff was not developed specifically for pregnant women, and a recent validation study has raised concerns about interpreting the FSFI total score in pregnant and postpartum populations [23]. Therefore, scores below the conventional threshold should be interpreted cautiously rather than as a definitive diagnosis of sexual dysfunction. Nevertheless, these findings highlight the importance of assessing sexual health as part of comprehensive antenatal care.

Lower urinary tract symptoms alone explained only a limited proportion of the variation in sexual function, whereas sociodemographic and obstetric factors contributed additional explanatory value. In the final model, greater severity of urinary filling symptoms was associated with lower FSFI scores. This finding is clinically relevant given the high prevalence of LUTS, particularly storage symptoms, during pregnancy [5]. However, sexual function during pregnancy is multifactorial and may be influenced by demographic, obstetric, psychological, and relationship-related factors in addition to physical symptoms [19,24]. Women without a history of curettage also had higher FSFI scores in the present study. Although curettage and abortion history are not equivalent, previous research has reported an association between a history of abortion and sexual dysfunction during pregnancy [25]. Therefore, the association observed in the present study should be interpreted cautiously and should not be considered evidence of a causal relationship between curettage and impaired sexual function. Overall, these findings highlight the importance of considering urinary symptoms together with maternal and obstetric history when assessing sexual health during pregnancy.

Gestational and obstetric characteristics were associated with both urinary symptoms and sexual function in the present study. The greater severity of filling symptoms in the third trimester is consistent with recent evidence showing that advanced gestation is associated with a greater burden of LUTS and that storage symptoms are particularly prominent in late pregnancy [5,26]. The associations of incontinence with gravidity and parity may reflect the cumulative effects of pregnancy and childbirth on pelvic floor function, although previous findings regarding parity are not entirely consistent. Sexual function also appears to vary according to gestational and obstetric characteristics, with recent studies reporting poorer sexual function as pregnancy progresses and highlighting the multifactorial nature of sexuality during pregnancy [25,27]. The lower sexual function observed among women with a history of curettage is also noteworthy, as previous research has identified a history of abortion as a potential factor associated with sexual dysfunction during pregnancy [25]. However, because curettage and abortion history are not equivalent constructs and the present study was cross-sectional, this finding should be interpreted cautiously. Overall, these results suggest that obstetric history may contribute differently to urinary and sexual health during pregnancy.

Pregnancy-related sexual characteristics were associated with both urinary symptoms and sexual function. Although greater sexual knowledge and more frequent intercourse were associated with higher filling and incontinence scores, these findings should be interpreted cautiously, as previous research has not consistently shown an association between intercourse frequency and urinary incontinence during pregnancy [28]. In contrast, the associations of intercourse frequency and sexual avoidance with several sexual function domains are consistent with evidence that sexual activity, desire, and sexual well-being commonly change during pregnancy [22,29]. Pregnancy-related concerns, discomfort, and fear of intercourse may contribute to sexual avoidance and poorer sexual function. Moreover, evidence that sexuality education during pregnancy can improve sexual function highlights the importance of appropriate counseling and sexual health assessment in antenatal care [30].

## 5. Strengths and Limitations

The strengths of this study include the use of validated instruments, such as the BFLUTS and FSFI, and the application of hierarchical multiple regression to assess the incremental contribution of different blocks of variables to the explained variance in sexual function. In addition, Bonferroni correction was applied to the relevant group comparisons to account for multiple testing and reduce the risk of Type I error.

Despite these strengths, this study has several limitations. First, due to the cross-sectional design, the temporal and causal direction of the associations between lower urinary tract symptoms and female sexual function could not be established. Second, although the final regression model was statistically significant, its modest explanatory power (adjusted R^2^ = 0.077) indicates that a substantial proportion of the variability in sexual function remains unexplained. Third, although 95.7% of the participants scored below the conventional FSFI cutoff of 26.55, this cutoff was developed for the general female population and has not been specifically established as a diagnostic threshold for pregnant women. Therefore, this proportion should be interpreted descriptively rather than as the clinical prevalence of female sexual dysfunction in the study population. The use of self-reported measures may also have introduced response bias. Furthermore, the single-center design, convenience sampling, requirement of being in a regular partner relationship, exclusion of first-trimester pregnant women, and predominance of third-trimester participants may have introduced selection bias and limit the generalizability of the findings to the broader pregnant population. Avoidance of sexual intercourse was assessed using a single question, which may not adequately capture the complexity of sexual avoidance behavior or the underlying reasons for avoidance. Finally, medication use was not specifically assessed; therefore, its potential influence on urinary symptoms and sexual function could not be accounted for in the analyses.

## 6. Conclusions

This study identified associations between urinary symptoms, obstetric history, and sexual function during pregnancy. Although correlations between individual urinary symptom and sexual function domains were generally limited, greater urinary filling symptom severity and a history of curettage were associated with lower overall sexual function scores in the adjusted regression model. Gestational trimester, gravidity, parity, and pregnancy-related sexual characteristics were also associated with variations in specific urinary symptom and sexual function domains.

These findings support the consideration of urinary symptoms, obstetric history, and sexual health as part of comprehensive antenatal assessment. Healthcare professionals may benefit from discussing urinary and sexual concerns with pregnant women and providing individualized counseling when needed. Prospective longitudinal studies are warranted to clarify the temporal relationships between urinary symptoms, obstetric characteristics, and sexual function throughout pregnancy.

## Figures and Tables

**Table 1:** Descriptive characteristics of the pregnant women (*n* = 278).

Characteristics	Mean	SD	Min–Max
Age	28.88	4.13	18–41
Spouse’ age	32.09	5.09	22–48
Year of marriage (year)	5.22	4.19	1–23
	***n***	**%**	
Educational status			
Middle Sch.	40	14.4	
High Sch.	80	28.8	
University and higher	158	56.8	
Employment status			
Not employed	173	62.2	
Employed	105	37.8	
Spouse educational status			
Middle Sch.	49	17.6	
High Sch.	116	41.7	
University and higher	113	40.6	
Spouse employment status			
Not employed	42	15.1	
Employed	236	84.9	
Income levels			
Middle	226	81.3	
High	52	18.7	
Family type			
Nuclear family	260	93.5	
Extended family	18	6.5	
Trimester			
Second trimester	71	25.5	
Third trimester	207	74.5	
Gravity			
Primigravida	110	39.6	
Multigravida	168	60.4	
Parity			
Nulliparous	145	52.2	
Primiparous	87	31.3	
Multiparous	46	16.5	
History of curettage			
Yes	47	16.9	
No	231	83.1	
Mode of delivery *			
Vaginal delivery	71	53.4	
Cesarean delivery	62	46.6	
Episiotomy *			
Yes	67	50.4	
No	66	49.6	
Vacuum/forceps-assisted delivery *			
Yes	5	3.8	
No	128	96.2	
Pregnancy-related sexual knowledge			
Yes	234	84.2	
No	44	15.8	
Pre-pregnancy sexual intercourse frequency			
Once a week	38	13.7	
Twice a week	153	55	
Three or more times per week	70	25.2	
Once every two weeks	17	6.1	
Intercourse frequency during pregnancy			
Once a week	89	32	
Twice a week	82	29.5	
Three or more times per week	13	4.7	
Once every two weeks	94	33.8	
Pre-pregnancy sexual satisfaction			
Dissatisfied	5	1.8	
Satisfied	273	98.2	
Avoidance of sexual intercourse during pregnancy			
Yes	121	43.5	
No	157	56.5	

SD: Standard Deviation. * Percentages do not total 100%, as women without a history of delivery after 20 weeks of gestation were excluded from this analysis.

**Table 2:** Descriptive Statistics of the BFLUTS, FSFI Scores and FSFI Cutoff Score.

Scales	Mean ± SD	Min–Max
The Bristol Female Lower Urinary Tract Symptoms Questionnaire-BFLUTS		
Filling	6.60 ± 2.40	1–13
Voiding	1.49 ± 1.73	0–7
Urinary Incontinence	2.83 ± 3.06	0–13
Sexual Function	0.44 ± 0.85	0–3
Quality of Life	4.06 ± 3.07	0–17
Female Sexual Function Index-FSFI		
Desire	3.95 ± 0.93	1.20–6
Arousal	3.22 ± 1.42	0–6
Lubrication	3.17 ± 1.44	0–5.70
Orgasm	3.08 ± 1.48	0–5.60
Satisfaction	2.07 ± 1.32	0–5.20
Pain	3.58 ± 1.86	0–6.00
Total	19.06 ± 5.72	2.70–30.40
	***n***	**%**
FSFI Cutoff Score		
FSFI ≥ 26.55, Normal sexual function	12	4.3
FSFI < 26.55, Female sexual dysfunction	266	95.7

SD: Standard Deviation.

**Table 3:** Pearson Correlations Between Female Sexual Function Index (FSFI) Subscale/Total Scores and BFLUTS Filling, Voiding, and Urinary Incontinence Subscale Scores With FDR-Adjusted *p* Values.

	FSFI						
	Desire	Arousal	Lubrication	Orgasm	Satisfaction	Pain	Total
BFLUTS							
Filling	r = −0.158, *p* = 0.008, q = 0.056	r = -0.129, *p* = 0.032, q = 0.168	r = 0.036, *p* = 0.547, q = 0.726	r = 0.047, *p* = 0.434, q = 0.726	r = −0.014, *p* = 0.821, q = 0.862	r = 0.042, *p* = 0.483, q = 0.726	r = −0.026, *p* = 0.669, q = 0.781
Voiding	r = 0.033, *p* = 0.580, q = 0.726	r = 0.196, *p* = 0.001, q = 0.021 *	r = 0.038, *p* = 0.531, q = 0.726	r = −0.094, *p* = 0.118, q = 0.413	r = 0.038, *p* = 0.530, q = 0.726	r = −0.161, *p* = 0.007, q = 0.056	r = −0.004, *p* = 0.940, q = 0.940
Urinary Incontinence	r = 0.043, *p* = 0.475, q = 0.726	r = −0.021, *p* = 0.731, q = 0.808	r = −0.035, *p* = 0.565, q = 0.726	r = −0.043, *p* = 0.473, q = 0.726	r = −0.033, *p* = 0.588, q = 0.726	r = −0.107, *p* = 0.076, q = 0.319	r = −0.060, *p* = 0.318, q = 0.726

r: Pearson correlation analysis, *p* = unadjusted *p* value; q = Benjamini–Hochberg false discovery rate (FDR)-adjusted *p* value. The FDR correction was applied across all 21 exploratory correlations. * q < 0.05.

**Table 4:** Results of Hierarchical Linear Regression Analysis for Total Female Sexual Function.

				Dependent Variable: Total Female Sexual Function (FSFI)								
	Variables	B	SE	β	t	*p*	95%CI	Tolerans-VIF	R^2^	ΔR^2^	F	*p*
Model 1	Filling	−0.033	0.151	−0.014	−0.217	0.828	−0.329–0.264	0.910−1.099	0.004	0.004	0.349	0.790
	Voiding	0.023	0.206	0.007	0.110	0.913	−0.383–0.428	0.943−1.061				
	Urinary Incontinence	−0.108	0.116	−0.058	−0.929	0.354	−0.337–0.121	0.937–1.068				
Model 2	Filling	−0.279	0.158	−0.117	−1.771	0.078	−0.590–0.031	0.777–1.287	0.107	0.103	2.246	0.007 *
	Voiding	0.162	0.209	0.049	0.777	0.438	−0.249–0.574	0.853–1.173				
	Urinary Incontinence	−0.083	0.120	−0.044	−0.690	0.491	−0.320–0.154	0.816–1.225				
	Age	−0.155	0.112	−0.112	−1.391	0.166	−0.375–0.065	0.523–1.912				
	Educational status (High Sch.)	−0.729	1.238	−0.058	−0.589	0.556	−3.166–1.708	0.353–2.834				
	Educational status (University and higher)	−1.582	1.181	−0.137	−1.340	0.181	−3.906–0.743	0.324–3.086				
	Income levels (High)	0.970	0.904	0.066	1.074	0.284	−0.809–2.749	0.892–1.121				
	Family type (Extended family)	2.383	1.405	0.103	1.696	0.091	−0.384–5.150	0.927–1.079				
	Year of marriage (year)	0.108	0.132	0.079	0.821	0.413	−0.151–0.368	0.365–2.741				
	Trimester (Third trimester)	0.904	0.805	0.069	1.123	0.262	−0.681–2.490	0.899–1.113				
	Gravity (Multigravida)	−0.095	1.144	−0.008	−0.083	0.934	−2.347–2.157	0.354–2.823				
	Parity (Multiparous)	−0.229	1.550	−0.015	−0.148	0.882	−3.280–2.822	0.334–2.993				
	Curettage experience (No)	2.983	1.078	0.196	2.766	0.006 *	0.860–5.107	0.678–1.475				
Model 3	Filling	−0.346	0.162	−0.145	−2.129	0.034 *	−0.666–−0.026	0.719–1.391	0.137	0.030	2.281	0.003 *
	Voiding	0.244	0.210	0.074	1.162	0.246	−0.169–0.657	0.831–1.204				
	Urinary Incontinence	−0.094	0.120	−0.050	−0.780	0.436	−0.330–0.143	0.808–1.237				
	Age	−0.156	0.112	−0.113	−1.395	0.164	−0.376–0.064	0.511–1.955				
	Educational status (High Sch.)	−0.780	1.232	−0.062	−0.633	0.527	−3.205–1.645	0.350–2.860				
	Educational status (University and higher.)	−1.308	1.187	−0.113	−1.102	0.271	−3.645–1.645	0.315–3.179				
	Income levels (High)	1.035	0.900	0.071	1.150	0.251	−0.737–2.808	0.882–1.133				
	Family type (Extended family)	1.686	1.414	0.073	1.192	0.234	−1.099–4.471	0.898–1.114				
	Year of marriage (year)	0.102	0.132	0.075	0.776	0.439	−0.157–0.361	0.359–2.784				
	Trimester (Third trimester)	0.942	0.818	0.072	1.152	0.251	−0.669–2.553	0.854–1.171				
	Gravity (Multigravida)	−0.620	1.149	−0.053	−0.540	0.590	−2.882–1.642	0.344–2.903				
	Parity (Multiparous)	0.025	1.538	0.002	0.016	0.987	−3.003–3.053	0.333–3.003				
	Curettage experience (No)	2.436	1.088	0.160	2.238	0.026 *	0.293–4.579	0.653–1.530				
	Pregnancy-related sexual knowledge (No)	−1.202	1.027	−0.077	−1.170	0.243	−3.225–0.821	0.773–1.293				
	Sexual intercourse frequency during pregnancy (Twice a week)	1.484	0.856	0.118	1.733	0.084	−0.202–3.170	0.713–1.403				
	Sexual intercourse frequency during pregnancy (Once every two weeks)	−0.711	0.831	−0.059	−0.856	0.393	−2.347–0.925	0.703–1.422				
	Sexual avoidance during pregnancy (No)	0.587	0.745	0.051	0.787	0.432	−0.881–2.054	0.796–1.256				

FSFI: Female Sexual Function Index. B: Unstandardized Coefficients, β: Standardized coefficients, SE: Std. Error, t: t-statistic, 95%CI: Confidence Interval for B, F: Model Statistics, R^2^: the proportion of explained variance in dependent variable by the model, ΔR^2^: the change in R^2^ values from one model to another. * *p* < 0.05. Model 1: The effect of Lower Urinary Tract Symptoms on Female Sexual Function, Model 2: The effect of demographic, obstetric and pregnancy-related sexual characteristics on Female Sexual Function, Model 3: The effect of all predictors on Female Sexual Function.

**Table 5:** Comparison of BFLUTS and FSFI Scores According to Obstetric and Pregnancy-Related Sexual Characteristics of The Pregnant Women.

	BFLUTS x⁻ ± SD			FSFI x⁻ ± SD						
Characteristics	Filling	Voiding	Urinary Incontinence	Desire	Arousal	Lubrication	Orgasm	Satisfaction	Pain	Total
Trimester										
Second trimester	5.59 ± 2.30	1.48 ± 1.81	2.76 ± 2.96	3.86 ± 1.06	3.30 ± 1.60	2.82 ± 1.64	2.90 ± 1.69	1.83 ± 1.28	3.45 ± 2.11	18.17 ± 5.67
Third trimester	6.95 ± 2.34	1.49 ± 1.70	2.86 ± 3.10	3.98 ± 0.89	3.19 ± 1.35	3.30 ± 1.35	3.14 ± 1.39	2.15 ± 1.33	3.62 ± 1.77	19.37 ± 5.72
	t = −4.231, *p* = 0.000 *, d = −0.584	t = −0.058, *p* = 0.954, d = −0.006	t = −0.235, *p* = 0.814, d = −0.033	t = −0.917, *p* = 0.360, d = −0.126	t = 0.561, *p* = 0.576, d = 0.084	t = −2.220, *p* = 0.029, d = −0.335	t = −1.054, *p* = 0.295, d = −0.159	t = −1.775, *p* = 0.077, d = −0.244	t = −0.594, *p* = 0.554, d = −0.089	t = −1.533, *p* = 0.128, d = −0.210
Gravity										
Primigravida	6.75 ± 2.13	1.79 ± 1.82	2.14 ± 2.86	3.88 ± 0.93	3.37 ± 1.36	3.30 ± 1.36	3.29 ± 1.45	2.36 ± 1.45	3.63 ± 1.76	19.82 ± 5.70
Multigravida	6.50 ± 2.56	1.49 ± 1.67	3.29 ± 3.11	4.00 ± 0.94	3.12 ± 1.45	3.09 ± 1.49	2.94 ± 1.48	1.88 ± 1.19	3.54 ± 1.93	18.56 ± 5.70
	t = 0.865, *p* = 0.388, d = 0.104	t = 0.013, *p* = 0.989, d = 0.000	t = −3.124, *p* = 0.002 *, d = −0.381	t = −1.034, *p* = 0.302, d = −0.127	t = 1.427, *p* = 0.155, d = 0.175	t = 1.198, *p* = 0.232, d = 0.147	t = 1.938, *p* = 0.054, d = 0.238	t = 2.990, *p* = 0.003 *, d = 0.367	t = 0.404, *p* = 0.687, d = 0.049	t = 1.800, *p* = 0.073, d = 0.221
Parity										
Nulliparous	6.56 ± 2.28	1.48 ± 1.84	2.09 ± 2.53	3.99 ± 0.96	3.40 ± 1.53	3.24 ± 1.48	3.12 ± 1.53	2.15 ± 1.44	3.54 ± 1.87	19.33 ± 5.59
Primiparous	6.36 ± 2.27	1.53 ± 1.70	3.11 ± 3.02	3.81 ± 0.92	2.83 ± 1.33	3.11 ± 1.42	2.95 ± 1.48	1.90 ± 1.23	3.60 ± 2.03	18.19 ± 6.15
Multiparous	7.20 ± 2.93	1.43 ± 1.42	4.65 ± 3.80	4.10 ± 0.83	3.38 ± 1.02	3.40 ± 1.36	3.18 ± 1.32	2.12 ± 1.05	3.68 ± 1.50	19.86 ± 5.19
	F = 1.900, *p* = 0.152, η^2^ = 0.014	F = 0.046, *p* = 0.955, η^2^ <.001	F = 13.946, *p* = 0.000 *, η^2^ = 0.092	F = 1.720, *p* = 0.181, η^2^ = 0.012	F = 4.977, *p* = 0.008, η^2^ = 0.035	F = 0.706, *p* = 0.495, η^2^ = 0.005	F = 0.514, *p* = 0.599, η^2^ = 0.004	F = 1.013, *p* = 0.364, η^2^ = 0.007	F = 0.104, *p* = 0.901, η^2^ = 0.001	F = 1.640, *p* = 0.196, η^2^ = 0.012
Curettage experience										
Yes	5.74 ± 2.35	1.87 ± 1.84	3.17 ± 3.19	4.34 ± 1.11	3.49 ± 1.84	2.47 ± 1.61	2.09 ± 1.59	1.39 ± 1.12	2.74 ± 2.14	16.52 ± 5.54
No	6.77 ± 2.38	1.41 ± 1.70	2.77 ± 3.04	3.87 ± 0.88	3.16 ± 1.31	3.32 ± 1.37	3.28 ± 1.37	2.21 ± 1.31	3.75 ± 1.75	19.58 ± 5.63
	t = −2.714, *p* = 0.007, d = −0.434	t = 1.675, *p* = 0.095, d = 0.267	t = 0.824, *p* = 0.411, d = 0.131	t = 2.745, *p* = 0.008, d = 0.512	t = 1.174, *p* = 0.245, d = 0.235	t = −3.380, *p* = 0.001 *, d = −0.602	t = −4.742, *p* = 0.000 *, d = −0.838	t = −4.004, *p* = 0.000 *, d = −0.641	t = −3.022, *p* = 0.004 *, d = −0.552	t = −3.401, *p* = 0.001 *, d = −0.544
Pregnancy-related sexual knowledge										
Yes	6.83 ± 2.36	1.51 ± 1.72	2.80 ± 3.16	3.91 ± 0.86	3.10 ± 1.34	3.27 ± 1.35	3.08 ± 1.36	2.05 ± 1.22	3.71 ± 1.82	19.12 ± 5.57
No	5.36 ± 2.63	1.39 ± 1.77	3.02 ± 2.53	4.17 ± 1.25	3.85 ± 1.63	2.67 ± 1.80	3.06 ± 1.10	2.15 ± 1.74	2.85 ± 1.95	18.77 ± 6.52
	t = 3.818, *p* = 0.000 *, d = 0.628	t = 0.430, *p* = 0.667, d = 0.069	t = −0.444, *p* = 0.658, d = −0.072	t = −1.344, *p* = 0.185, d = −0.285	t = −2.895, *p* = 0.005, d = −0.543	t = 2.086, *p* = 0.042, d = 0.418	t = 0.048, *p* = 0.962, d = 0.010	t = −0.371, *p* = 0.712, d = −0.077	t = 2.843, *p* = 0.005, d = 0.467	t = 0.331, *p* = 0.742, d = 0.061
Pre-pregnancy sexual intercourse frequency										
Once a week	7.21 ± 2.38	1.97 ± 1.97	3.87 ± 2.95	4.09 ± 0.98	3.43 ± 1.98	2.89 ± 1.76	2.65 ± 1.79	1.93 ± 1.47	2.91 ± 2.21	17.89 ± 6.50
Twice a week	6.18 ± 2.28	1.33 ± 1.56	2.56 ± 3.22	4.04 ± 0.86	3.31 ± 1.38	3.21 ± 1.38	3.18 ± 1.49	2.12 ± 1.31	3.58 ± 1.82	19.42 ± 5.77
Three or more times per week	7.41 ± 2.47	1.43 ± 1.71	2.76 ± 2.78	3.63 ± 0.10	2.85 ± 1.11	3.22 ± 1.30	3.09 ± 1.17	1.83 ± 0.95	3.85 ± 1.57	18.48 ± 4.51
Once every two weeks	5.71 ± 2.09	2.12 ± 2.37	3.29 ± 2.62	4.16 ± 0.94	3.39 ± 1.22	3.17 ± 1.44	3.06 ± 1.61	2.96 ± 1.89	3.98 ± 2.23	20.85 ± 7.49
	F = 6.216, *p* = 0.000 *, η^2^ = 0.064	F = 2.259, *p* = 0.082, η^2^ = 0.024	F = 2.011, *p* = 0.113, η^2^ = 0.022	F = 3.794, *p* = 0.011, η^2^ = 0.040	F = 2.131, *p* = 0.97, η^2^ = 0.023	F = 0.593, *p* = 0.620, η^2^ = 0.006	F = 1.285, *p* = 0.280, η^2^ = 0.014	F = 3.728, *p* = 0.012, η^2^ = 0.039	F = 2.437, *p* = 0.065, η^2^ = 0.026	F = 1.543, *p* = 0.204, η^2^ = 0.017
Intercourse frequency during pregnancy										
Once a week	6.62 ± 2.56	1.48 ± 1.55	2.11 ± 2.67	3.80 ± 0.10	3.25 ± 1.77	3.03 ± 1.61	3.09 ± 1.56	2.14 ± 1.32	3.33 ± 1.88	18.64 ± 6.29
Twice a week	6.91 ± 2.37	1.23 ± 1.70	3.26 ± 3.15	3.80 ± 0.74	3.20 ± 0.97	3.53 ± 0.82	3.62 ± 0.67	2.31 ± 1.03	4.27 ± 1.16	20.74 ± 2.37
Three or more times per week	6.46 ± 1.76	1.15 ± 1.52	6.38 ± 4.33	4.25 ± 1.33	2.68 ± 0.87	3.07 ± 1.14	2.83 ± 0.92	1.66 ± 0.78	4.22 ± 1.43	18.70 ± 4.49
Once every two weeks	6.33 ± 2.34	1.77 ± 1.91	2.66 ± 2.78	4.19 ± 0.91	3.27 ± 0.87	3.01 ± 1.68	2.63 ± 1.80	1.85 ± 1.54	3.11 ± 2.18	18.05 ± 6.99
	F = 0.882, *p* = 0.451, η^2^ = 0.010	F = 1.587, *p* = 0.193, η^2^ = 0.017	F = 8.773, *p* = 0.000 *, η^2^ = 0.088	F = 4.143, *p* = 0.007, η^2^ = 0.043	F = 0.685, *p* = 0.562, η^2^ = 0.007	F = 2.404, *p* = 0.068, η^2^ = 0.026	F = 7.196, *p* = 0.000 *, η^2^ = 0.073	F = 2.365, *p* = 0.071, η^2^ = 0.025	F = 7.293, *p* = 0.000 *, η^2^ = 0.074	F = 3.605, *p* = 0.014, η^2^ = 0.038
Avoidance of sexual intercourse during pregnancy										
Yes	6.60 ± 2.46	1.62 ± 1.77	2.71 ± 2.80	4.35 ± 0.98	3.47 ± 1.45	2.84 ± 1.58	2.68 ± 1.77	1.89 ± 1.52	2.84 ± 2.10	18.07 ± 6.32
No	6.60 ± 2.36	1.39 ± 1.69	2.93 ± 3.25	3.64 ± 0.77	3.02 ± 1.36	3.43 ± 1.29	3.38 ± 1.12	2.21 ± 1.12	4.15 ± 1.41	19.83 ± 5.10
	t = 0.016, *p* = 0.987, d = 0.000	t = 1.108, *p* = 0.269, d = 0.133	t = −0.602, *p* = 0.547, d = −0.072	t = 6.639, *p* = 0.000 *, d = 0.828	t = 2.691, *p* = 0.008, d = 0.326	t = −3.383, *p* = 0.001 *, d = −0.421	t = −3.841, *p* = 0.000 *, d = −0.491	t = −1.956, *p* = 0.052, d = −0.246	t = −5.912, *p* = 0.000 *, d = −0.751	t = −2.501, *p* = 0.013, d = −0.311

t = independent-samples *t*-test; F = one-way ANOVA; d = Cohen’s d; η^2^ = eta squared. A Bonferroni-adjusted significance threshold of *p* < 0.005 (0.05/10) was applied to the 10 outcome comparisons within each characteristic. For significant ANOVA results, pairwise comparisons were performed using the Bonferroni post hoc test; different superscript letters indicate significant pairwise differences (Bonferroni-adjusted *p* < 0.05). * *p* < 0.005.

## Data Availability

Data are available from the corresponding author upon reasonable request, subject to ethical and privacy restrictions.

## Abbreviations

The following abbreviations are used in this manuscript:

BFLUTS	Bristol Female Lower Urinary Tract Symptoms Questionnaire
FSFI	Female Sexual Function Index
LUTS	Lower Urinary Tract Symptoms
